# Intermediate Range Order in Metal–Ammonia Solutions: Pure and Na-Doped Ca-NH_3_

**DOI:** 10.1021/acs.jpcb.1c03843

**Published:** 2021-07-02

**Authors:** Thomas C. Nicholas, Thomas F. Headen, Jonathan. C. Wasse, Christopher. A. Howard, Neal. T. Skipper, Andrew G. Seel

**Affiliations:** † Department of Chemistry, Inorganic Chemistry Laboratory, University of Oxford, South Parks Road, Oxford OX1 3QR, U.K.; ‡ ISIS Spallation Neutron and Muon Source, STFC Rutherford Appleton Laboratory, Didcot, Oxfordshire OX11 0QX, U.K.; § Department of Physics and Astronomy, University College London, Gower Street, London WC1E 6BT, U.K.

## Abstract

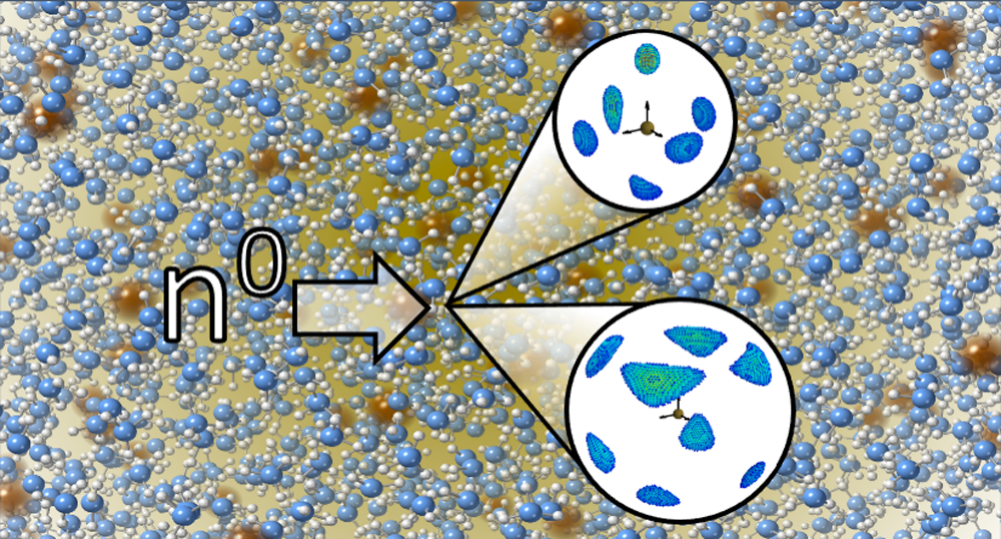

The local and intermediate range ordering in Ca–NH_3_ solutions in their metallic phase is determined through H/D isotopically differenced neutron diffraction in combination with empirical potential structure refinements. For both low and high relative Ca concentrations, the Ca ions are found to be octahedrally coordinated by the NH_3_ solvent, and these hexammine units are spatially correlated out to lengthscales of ∼7.4–10.3 Å depending on the concentration, leading to pronounced ordering in the bulk liquid. We further demonstrate that this liquid order can be progressively disrupted by the substitution of Ca for Na, whereby a distortion of the average ion primary solvation occurs and the intermediate range ion–ion correlations are disrupted.

## Introduction

The dissolution of electropositive group I and group II metals in liquid NH_3_ yields a rich series of electronic liquids, whose properties are chemically tunable through both the concentration and nature of the parent metal.^1,2^ Ca, as a divalent metal, shares many of the fascinating properties commonly associated with the group I metals when dissolved in NH_3_, forming an electrolytic system of solvated electrons at low concentrations and itinerant, metallic liquids as the concentration of Ca is increased.^1,3^ As with other metal–ammonia systems, these different electronic states are not miscible and are separated by a forbidden region around a metal–insulator transition whereby a bulk liquid–liquid phase separation occurs, commonly referred to as the immiscibility region in the literature. Unusually, this immiscibility region is extremely pronounced in the Ca–NH_3_ phase diagram, with the electrolytic state only surviving to a maximum Ca concentration of 1.68 mole percent metal (MPM) at the consolute point or a critical temperature of 290 K with the onset of phase separation at <0.1 MPM at 200 K.^1,4^ For comparison, the Na–NH_3_ consolute point is 4.12 MPM and 231 K with phase separation beginning at 1 MPM at 220 K, and in Li–NH_3_, the consolute point is 4.35 MPM at 210 K with phase separation beginning at 2 MPM at 220 K.^5^ Such early onset and pronounced liquid–liquid phase separation clearly indicate the stability of, or energetic drive toward, the metallic liquid in Ca–NH_3_ solutions.

Previous studies have sought to understand the link between the different electronic states of metal–ammonia systems and their liquid structure, whereby the charge of the metal cation, the extent of screening by the itinerant electron, and the secondary solvation effects from the NH_3_ solvent can all be varied. Some metal–ammonia systems, including Li–NH_3_ and Ca–NH_3_, have been shown to possess strong intermediate range ordering between ∼6 and 10.5 Å in their metallic phases, whereas other systems such as Na–NH_3_ and K–NH_3_ do not.^6−9^ Interestingly, only those systems which exhibit such strong ordering in their liquid state are known to crystallize as solid phases at their concentration limit. All other metal–ammonia or metal–amine systems precipitate out the metal upon cooling and do not form solid metal–amine phases. Taking only the examples of the Ca–NH_3_ and Na–NH_3_ systems, it is notable that, despite their similar ionic-radii,^10^ Ca–NH_3_ possesses distinct intermediate range ordering in the liquid and crystallizes as the expanded metal Ca(NH_3_)_6_,^9,11^ whereas no such liquid order nor any crystalline phases have been witnessed in Na–NH_3_.^8^

Herein, we use neutron scattering with combined H/D isotopic substitutions and empirical-potential structure refinements^12^ to provide a more detailed study of the Ca–NH_3_ system in the metallic phase than has been performed previously. Both the local and intermediate solvation environments of the Ca ions are determined alongside Ca–Ca correlations in the liquid phase. We also demonstrate that the intermediate range order of the Ca–NH_3_ solution can be successively disrupted by the introduction of Na.

## Experimental Methods

Stoichiometric amounts of mechanically cleaned metal were loaded under argon into a sealed flat-plate null-coherent scattering Ti/Zr container of size 15 mm × 30 mm × 1 mm. The container and sample were attached to a stainless steel gas rig and evacuated to <10^–5^ mbar. Appropriate ratios of isotopically unique ammonia (NH_3_/ND_3_) were premixed to the exact volume required and cryogenically pumped onto the metal samples. The samples were isolated, warmed, and monitored for homogeneity. No notable sample decomposition occurred as monitored by lack of H_2_ evolution.

H/D substitution experiments on the Ca–NH_3_ and mixed metal NaCa–NH_3_ solutions were performed using the SANDALS and NIMROD diffractometers, respectively, at the ISIS Spallation Neutron and Muon Source, UK.^13,14^

The sample data were corrected for sample containment, multiple scattering, and incoherent scattering according to standard procedures using the Gudrun package.^15,16^ The resultant total structure factor can be expressed as
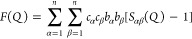
1where *c*_*i*_ and *b*_*i*_ are the atomic fraction and bound coherent neutron scattering length of species *i*, respectively. *S*_αβ_(*Q*) is the Faber–Ziman partial structure factor, and *Q* is the magnitude of the scattering vector.

The real space function *G*(*r*) corresponding to *F*(*Q*) is obtained by replacing *S*_αβ_(*Q*) with the partial pair distribution functions *g*_αβ_(*r*) by the signed Fourier transform relationship to give
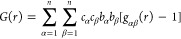
2

In the current context, *G*(*r*) can be written as a sum of three partial pair distribution functions
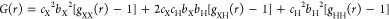
3where the composite coherent scattering length *b*_X_ and atomic concentration *c*_X_ are defined as
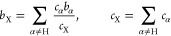
4

The label H refers to substituted atoms in the sample, and X refers to unsubstituted atomic species. It follows from eq 4 that *c*_H_ = 1 – *c*_X_. Then, the *g*_HH_(*r*) term in eq 3 is calculated from
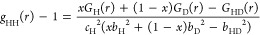
5where the subscripts H, D, and HD refer to experiments on the protonated, deuterated, and mixture samples, respectively, and *x* is the fraction of protonated ammonia in the mixture sample. Then
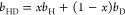
6

The remaining partial pair distribution functions can be calculated in an analogous way to *g*_HH_(*r*) – 1 in eq 3
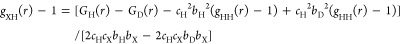
7
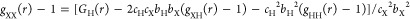
8

Structural models for the liquid systems at 4 and 10 MPM were refined to the respective neutron datasets using the empirical potential structure refinement (EPSR) software.^12^ This technique refines a structural model based upon an equilibrated Monte Carlo simulation using seed pair-potentials. These potentials are iteratively modified through the introduction of an empirical potential based upon the difference between simulated and measured, isotopically unique neutron scattering structure factors. Simulation boxes consisted of 6000 molecules within the cubic cell of length 64.588 and 66.674 Å for the 4 and 10 MPM systems, respectively, constructed using the reported densities for the Ca–NH_3_ system. The seed Lennard-Jones plus Coulomb potentials used in the refinement are reported in Table 1. The all-atom optimized potential for liquid simulation Lennard-Jones parameters of Jorgensen have been used for NH_3_ with an adjustable Coulomb term to ensure net neutrality in the simulations of the itinerant electron systems.^17^ The reduced charge of Ca ions was taken as those reported from *ab initio* simulations for single ions in NH_3_ solutions^18^ and was found to be necessary for subsequent structural refinement to the experimental neutron data.

**Table 1:** Lennard-Jones Plus Coulomb Parameters for Seed Potentials Used in EPSR Refinements

atom	σ_αα_ (Å)	ϵ_αα_ (kJ mol–1)	*q* (e)
N	3.420	0.711	–1.020
H	0.0	0.0	0.34–*y*^a^
Ca	2.412	1.380	1.530

a *y* is introduced to ensure a net-neutral system, defined as *N*_Ca_*q*_Ca_/3*N*_NH3_ where *N*_*i*_ is the number of species *i*.

## Results and Discussion

**Figure 1:**
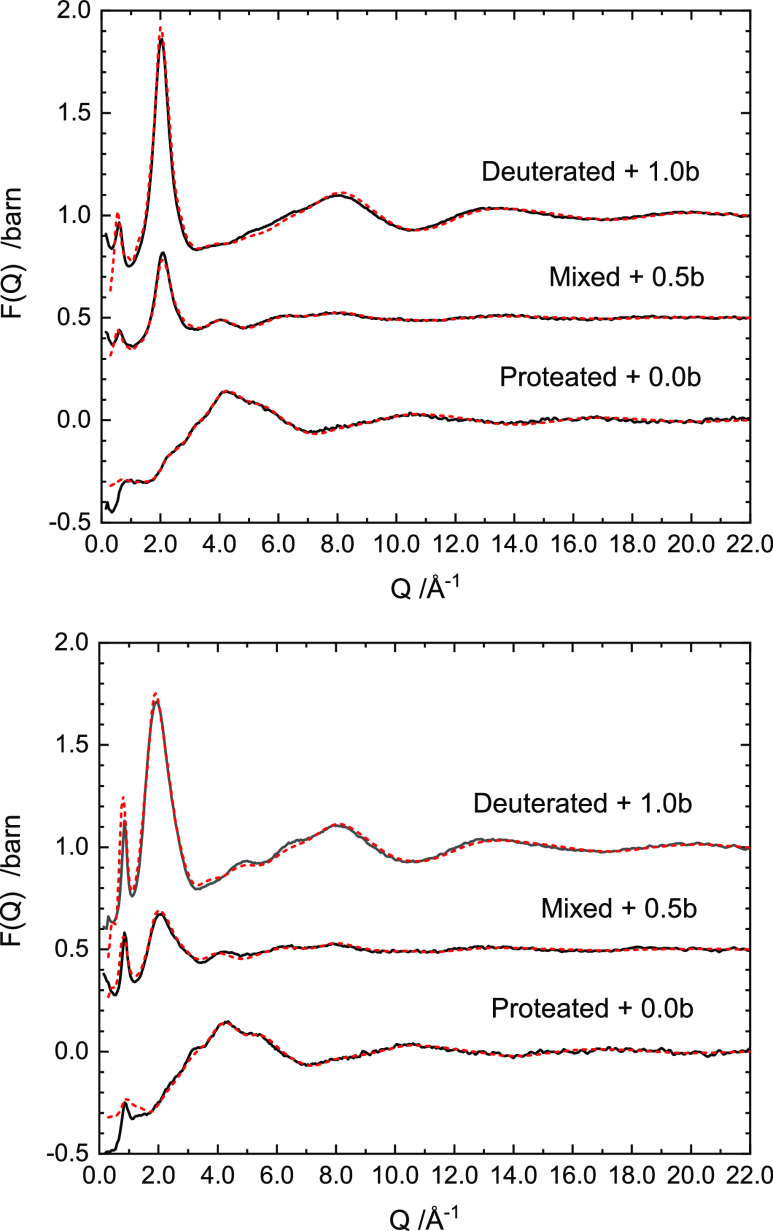
Total structure factor *F*(*q*) data for isotopically unique Ca–NH_3_ samples at concentrations 4 MPM (top) and 10 MPM (bottom). Data are offset by a given amount for clarity. From top to bottom: deuterated, mixed, and proteated. EPSR refinements to the data are shown by dashed red lines.

The experimental neutron total structure factors, *F*(*Q*), for metallic Ca–NH_3_ solutions at 4 and 20 MPM are presented in Figure 1 for the deuterated, proteated, and mixed proteated–deuterated samples. The sharp, concentration-dependent prepeak at the lowest *Q*-values is characteristic of those metallic solutions which possess a strong intermediate range order of the solvated Ca^2+^ cationic centers with increasing metal content. Their occurrence at 0.61 and 0.85 Å^–1^ indicate Ca–Ca correlations at ∼7.4 and ∼10.3 Å in the 10 and 5 MPM solutions, respectively. Our measurements are thus in agreement with previous neutron measurements of Ca–NH_3_.^6,9^ It can also be seen that the principal diffraction peak in the 2.5–3.0 Å^–1^ region displays considerable broadening with increasing metal concentration, and the feature between 4.0 and 10.0 Å^–1^ is distinctly structured in the more concentrated Ca–NH_3_ solution.

**Figure 2:**
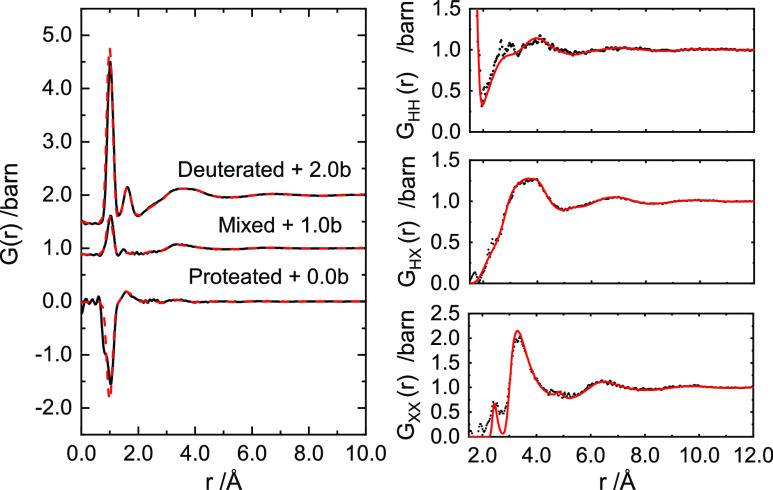
Left: Real space function *G*(*r*) for isotopically unique 4 MPM Ca–NH_3_ samples. Data are offset by a given amount for clarity. From top to bottom: deuterated, mixed, and proteated. Right: Partial pair distribution function data for 4 MPM Ca–NH_3_. From top to bottom: *G*_HH_(*r*), *G*_HX_(*r*), and *G*_XX_(*r*). EPSR refinements to the data are shown by red lines.

**Figure 3:**
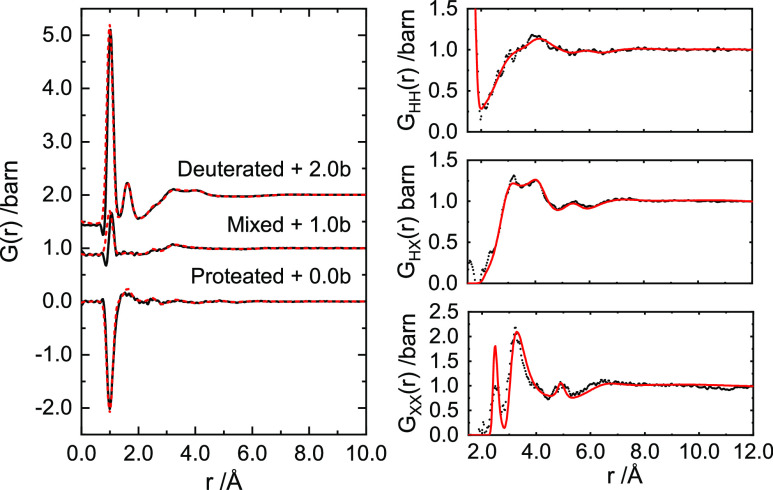
Left: Real space function *G*(*r*) for isotopically unique 10 MPM Ca–NH_3_ samples. Data are offset by a given amount for clarity. From top to bottom: deuterated, mixed, and proteated. Right: Partial pair distribution function data for 10 MPM Ca–NH_3_. From top to bottom: *G*_HH_(*r*), *G*_HX_(*r*), and *G*_XX_(*r*). EPSR refinements to the data are shown by red lines.

The origin of these features is clearer when looking at the real space transforms for the neutron data. Figures 2 and 3 show the real space correlation functions, *G*(*r*), for the 4 and 10 MPM Ca–NH_3_ data, respectively, as well as the extracted partial pair distribution functions, *G*_HH_(*r*), *G*_XH_(*r*), and *G*_XX_(*r*) as defined in eqs 5, 7, and 8. It can be seen that the *G*_XH_(*r*) and *G*_XX_(*r*) partials are far more structured in the 10 MPM Ca–NH_3_ solution than they are at 4 MPM in the 2.5–6.0 Å region, indicating a distinctly ordered solvation environment of the Ca ions. In particular, we can assign the feature at 2.52 Å in *G*_XX_(*r*) to the Ca–N distance, being far more pronounced in the 10 MPM solution. Likewise, the narrowing in the most intense feature to 3.2 Å and the appearance of a peak at 5.0 Å can be assigned to *cis*- and *trans*-*N*–*N* distances, which would be consistent with an octahedral arrangement of NH_3_ around Ca. It is interesting to compare these liquid, metallic Ca–NH_3_ solutions to solvated, aqueous calcium solutions,^19^ whereby the Ca–O distances reported are only slightly shorter at 2.46 Å despite the aqueous ions being 8-coordinate as opposed to 6-coordinate for the amines. Turning to the *G*_XH_(*r*) partials, we see again that the 10 MPM system is more structured with correlations presumably being successive Ca–H, N–H distances.

**Figure 4:**
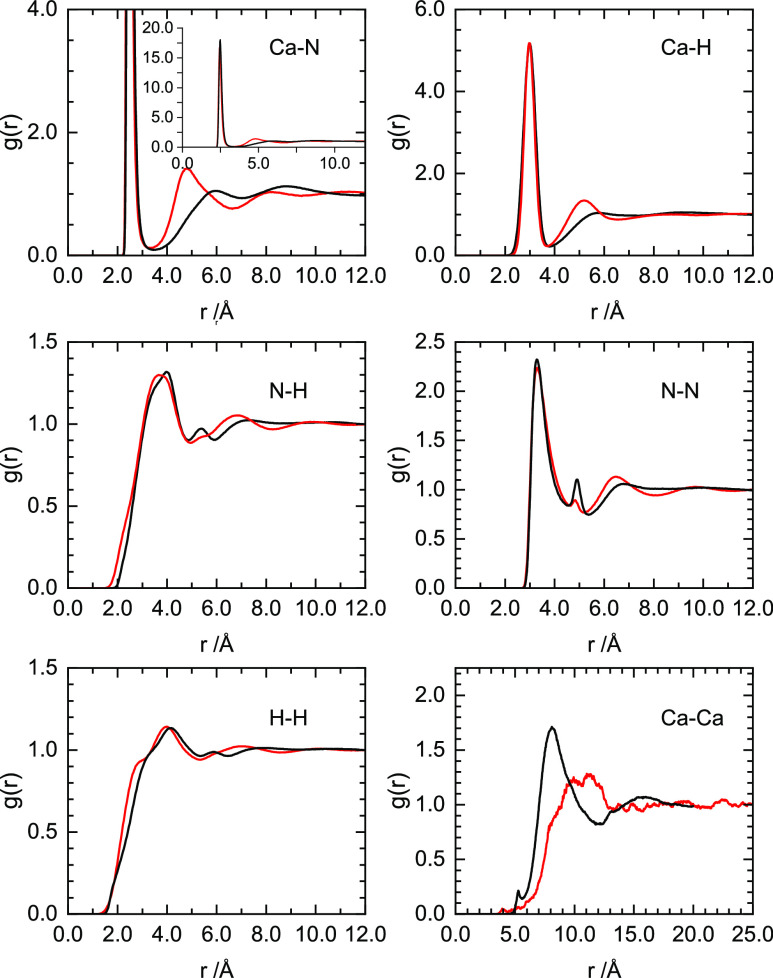
Extracted partial pair radial distribution functions for 4 MPM (red) and 10 MPM (black) Ca–NH_3_ solutions. Column 1, from top to bottom: *g*_CaN_(*r*), *g*_NH_(*r*), and *g*_HH_(*r*). Column 2, from top to bottom: *g*_CaN_(*r*), *g*_NH_(*r*), and *g*_HH_(*r*).

In order to extract more information from the experimental datasets, individual pair distribution functions were determined from EPSR models for both 4 and 10 MPM systems, with refinements reported alongside the experimental data in Figures 1–3. These structural models, refined as they are to the isotopically unique neutron datasets, are a notable improvement on previous Monte Carlo simulations for the Ca–NH_3_ solutions.^9^ The refinements for both 4 and 10 MPM solutions are well described even down to the low-*Q* region where the relative intensities of the prepeak in *F*(*Q*) are matched across datasets, albeit slightly over-fit in the 10 MPM system. Likewise, the full extent of the structuring of the feature between 4.0 and 10.0 Å^–1^ in the more concentrated Ca–NH_3_ solution is not fully captured by the EPSR model. We can see clearly where this discrepancy arises by examining the simulated *G*_XX_(*r*). The Ca–N distance is correct in the EPSR refinement, but the feature is sharper than in the measured neutron data, where the intensity is moved into the peak wings. This is most likely due to the insufficiencies of the seed and empirical potentials being too “hard” for a good description of the Ca ions in a metallic solution. Nevertheless, the high level of agreement between our structural models and the neutron data lends credence to our extracted pair distributions functions presented in Figure 4.

The pair distribution functions extracted from the EPSR model allow us to confirm our assignment of the Ca–N and N–N distances from the measured neutron data and the origin of the prepeak as being due to Ca–Ca correlations. The structuring of the measured *G*_XH_(*r*) partials in the 10 MPM solution can now be interpreted further as the increasing contribution from the Ca–H correlations at 3.0 Å as the Ca concentration is increased. Only a minor low-*r* shoulder is seen in the N–H partials, in agreement with the large disruption of any hydrogen bonding in these metallic systems, a commonality of all measured metal–amine systems in their metallic phases. The EPSR models also allow us to determine correlations which are only a minor contribution to the experimental data, such as the secondary solvation of Ca(NH_3_)_6_ in the 4 MPM solution. Clear secondary and tertiary solvation distances are found at 4.8 and 8.2 Å.

**Figure 5:**
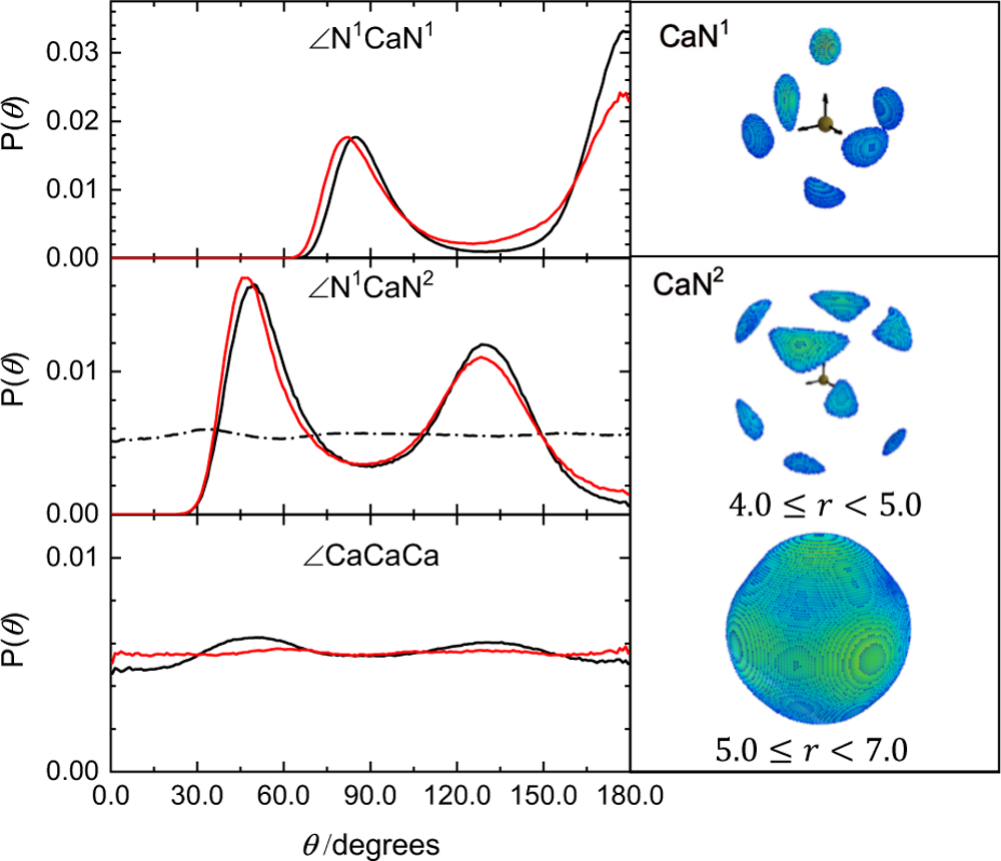
Left: Angular probability densities for 4 MPM (red) and 10 MPM (black) Ca–NH_3_ solutions. Labels indicate primary solvation environment around Ca (N^1^, 2 ≤ *r* ≤ 3 Å), secondary solvation (N^2^, 4 ≤ *r* ≤ 7 Å) where the full and dashed lines for the 10 MPM system are integrated in the ranges 4 ≤ *r* ≤ 5 and 5 ≤ *r* ≤ 7 Å, respectively, and Ca ion–ion orientations. Right: Corresponding spatial density maps for N atoms around a central Ca ion in the 10 MPM system.

In order to build up a three-dimensional picture of the Ca–NH_3_ system, Figure 5 details the angular distributions and spatial probability density maps extracted from the refined structural models for Ca–NH_3_ solutions. As expected from our assignments of the experimental data, the inner coordination sphere (corresponding to the sharp first peak in *g*_CaN_(*r*) in Figure 4) shows an octahedral arrangement of ammonia nitrogen atoms about the central Ca. This solvation is more regular for the 10 MPM system, with the 4 MPM system having a slightly less well-defined octahedral geometry as demonstrated with the broadening of angular distributions for NH_3_ coordinated trans to each other. The second coordination shell (corresponding to the broader *g*_CaN_(*r*) peak in the range of 4.0–7.0 Å) contains those NH_3_ molecules not directly bound to the Ca ions in solution and demonstrate a drastic change between those located up to ∼5 Å and those beyond. The second peak in *g*_CaN_(*r*) for the 4 MPM solutions occurs at 4.8 Å, where there is only a minor contribution in the 10 MPM system. This can be assigned to the hydrogen-bonded, secondary solvation of the Ca centers and can be seen in Figure 5 corresponding to a facial approach to the primary Ca(NH_3_)_6_ species. The 10 MPM system exhibits its secondary solvation maximum in *g*_CaN_(*r*) at 5.9 Å, at which distance there is very little angular dependence in the spatial density. This demonstrates that neighboring Ca(NH_3_)_6_ units are only weakly correlated in terms of their relative orientations despite the strong radial correlation between Ca ions in the metallic solutions. While in the 4 MPM solutions, there is no angular correlation in the distribution of Ca ions, in the 10 MPM solution, we do find a slight dominance for a facial approach of one Ca(NH_3_)_6_ unit to the next, consistent with the *bcc* arrangement of the solid calcium hexammine structure, Ca(NH_3_)_6_, formed at the pseudo-eutectic point whereby cooling any metallic Ca–NH_3_ solution to 185 K excess NH_3_ crystallizes out leaving only the expanded metal.^20,21^ This expanded-metal hexammine crystallizes in the *Im*3*m* space group with a lattice constant, *a*_o_ = 9.12 Å, and an octahedral orientation of NH_3_ molecules around each Ca atom.^11,20^ Bond distances in the crystal have been determined as *r*(Ca–N) = 2.56 Å and *r*(Ca–D) = 3.12 Å, correlations which we have now shown to persist into the liquid state, even upon further dilution by additional NH_3_ as in the 4 MPM solution.

We have previously suggested that for a metal–amine system to crystallize into an expanded metal upon cooling, there must be a substantial order already present in the liquid both in terms of primary solvation and intermediate range order of the metal centers. This leads to the stability of the pseudo-eutectic and prevents the formation of crystalline NH_3_ and metal. Our findings for the Ca–NH_3_ system further strengthen this viewpoint and certainly provide a solution-phase limit in the future search for new, crystalline expanded metals.

We can investigate the manner in which the structural order in the liquid Ca–NH_3_ system can be disrupted by examining the effect of progressive substitution of Ca by Na. The ionic radii of Ca^2+^ and Na^+^ are similar, and both have been determined to be octahedrally coordinated in aqueous salt solutions, but in the case of Na^+^, there is some uncertainty in this, with the experimentally determined coordination numbers ranging from 4 to 8.^22,23^ We should also note that when considering NaCa–NH_3_ solutions in their metallic phase, despite maintaining the concentration of metal ions (MPM), the density of conduction electrons in the solution (sometimes referred to as mole-percent electrons) can vary up to a factor of 2 due to the difference in valence between Na and Ca. This conduction electron density is believed to lie predominantly between solvated ion units in metal–amine solutions, and we can thus determine the effect on the liquid structure of varying this electron density through control of the Na/Ca content.

**Figure 6:**
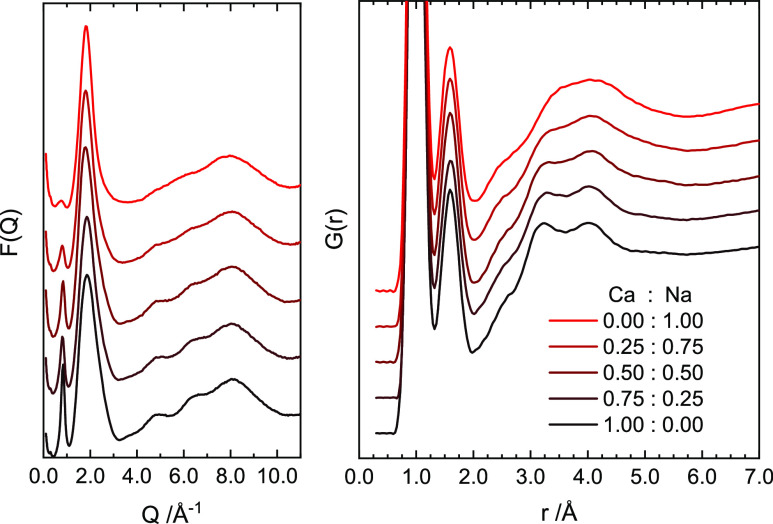
Left: Neutron total structure factor *F*(*q*) data for Na-doped 10 MPM Ca–NH_3_ solutions. Data are offset for clarity. Right: Corresponding *G*(*r*) data.

Figure 6 reports the experimental neutron structure factors for the 10 MPM, deuterated NaCa–NH_3_ system at various Na/Ca ratios. We can clearly see that the pronounced prepeak in Ca–NH_3_ rapidly decreases in intensity as the Na content increases, indicating that ion–ion correlations are decreasing, and both the width of the principal peak and structuring of the feature from 4.0 to 10.0 Å^–1^ decrease. This would indicate a disruption of the octahedral arrangement of the solvated metal ions, which can be confirmed by examining the equivalent data in real space. Interestingly, while the M–NH_3_ correlations are still found at ∼2.5 Å, we see a slight shift to shorter bond lengths as the Na content increases, despite the lower charge of these ions, and there is a progressive disruption of the structure in the 3.0–5.0 Å range. This shorter bond is consistent with the Na–N distances previously reported for pure Na–NH_3_ solutions in their metallic phase.^8^ It is likely that the longer Ca–N distances in Ca–NH_3_ is a reflection of the highly structured Ca(NH_3_)_6_ units being more “molecular” in nature than the Na–NH_3_ analogue. It is clear that the incorporation of Na ions into the Ca–NH_3_ solution disrupts the local solvation structure of the metal ions, with the distinctly octahedral coordination of Ca being lost upon substitution by Na. This in turn leads to a loss of intermediate range order in the mixed-ion solutions.

## Conclusions

Despite its molecular nature and the large concentration range over which a metallic phase occurs, the liquid Ca–NH_3_ system is structurally quite simple. There is a strong local ordering whereby the octahedral coordination of Ca by NH_3_ comprise the solvated ion cores for both the most dilute and more concentrated itinerant electron liquids. The approach of additional NH_3_ is toward the octahedral faces, being driven by the formation of hydrogen bonding interactions with the solvated ion cores. Beyond this, further NH_3_ molecules still exist in distinct solvation shells but are spatially isotropic. It is striking to note that even at low metal concentrations of 4 MPM, a significant intermediate range order exists in these solutions, with the solvated ion cores being correlated out to >10 Å. This becomes more pronounced and with a shift to a shortened distance of 7.4 Å for the 10 MPM solution. As we have previously noted, this structuring in the liquid state seems to be a feature of metal–amine systems, which undergo crystallization at their concentration limit, and is significantly reduced or absent in those which do not.^24^

Furthermore, we have showed that the miscibility of Na– and Ca–NH_3_ enables the local and intermediate structure of the bulk liquid to be controlled as a function of the conduction electron density. Sequential disruption of the primary solvation environment upon incorporation of Na is evident from the neutron scattering data, which in turn is reflected in the intermediate range, ion–ion correlations being reduced. Whether this has an effect on the electronic properties of the liquids, or indeed one which can be carried across into any crystalline material, is yet to be determined. It is now clear that, at the very least, the liquid structure for metal–NH_3_ and other metal–amine systems is amenable to chemical control.
